# Circulating small non-coding RNAs reflect IFN status and B cell hyperactivity in patients with primary Sjögren’s syndrome

**DOI:** 10.1371/journal.pone.0193157

**Published:** 2018-02-15

**Authors:** Ana P. Lopes, Maarten R. Hillen, Eleni Chouri, Sofie L. M. Blokland, Cornelis P. J. Bekker, Aike A. Kruize, Marzia Rossato, Joel A. G. van Roon, Timothy R. D. J. Radstake

**Affiliations:** 1 Department of Rheumatology & Clinical Immunology, University Medical Center Utrecht, Utrecht University, Utrecht, the Netherlands; 2 Laboratory of Translational Immunology, University Medical Center Utrecht, Utrecht University, Utrecht, the Netherlands; 3 Functional Genomics Center, University of Verona, Verona, Italy; University of Massachusetts Medical School, UNITED STATES

## Abstract

**Background:**

Considering the important role of miRNAs in the regulation of post–transcriptional expression of target genes, we investigated circulating small non-coding RNAs (snc)RNA levels in patients with primary Sjögren’s syndrome (pSS). In addition we assessed if serum sncRNA levels can be used to differentiate patients with specific disease features.

**Methods:**

Serum RNA was isolated from 37 pSS patients as well as 21 patients with incomplete Sjögren’s Syndrome (iSS) and 17 healthy controls (HC) allocated to two independent cohorts: discovery and validation. OpenArray profiling of 758 sncRNAs was performed in the discovery cohort. Selected sncRNAs were measured in the validation cohort using single-assay RT-qPCR. In addition, unsupervised hierarchical clustering was performed within the pSS group.

**Results:**

Ten sncRNAs were differentially expressed between the groups in the array. In the validation cohort, we confirmed the increased expression of U6-snRNA and miR-661 in the iSS group as compared to HC. We were unable to validate differential expression of any miRNAs in the pSS group. However, within this group several miRNAs correlated with laboratory parameters. Unsupervised clustering distinguished three clusters of pSS patients. Patients in one cluster showed significantly higher serum IgG, prevalence of anti-SSB autoantibodies, IFN-score, and decreased leukocyte counts compared to the two other clusters.

**Conclusion:**

We were unable to identify any serum sncRNAs with differential expression in pSS patients. However, we show that circulating miRNA levels are associated with disease parameters in pSS patients and can be used to distinguish pSS patients with more severe B cell hyperactivity. As several of these miRNAs are implicated in the regulation of B cells, they may play a role in the perpetuation of the disease.

## Introduction

Primary Sjögren’s syndrome (pSS) is a systemic chronic autoimmune disease characterized by lymphocytic infiltration of salivary and lacrimal glands, associated with dryness of mouth (xerostomia) and eyes (keratoconjunctivitis sicca). pSS patients may present with extra-glandular manifestations such as renal, pulmonary or neurologic involvement and around 5% of the patients develop lymphoma, primarily of the mucosa-associated lymphoid tissue (MALT) [1, 2]. B cell hyperactivity is one of the hallmarks of pSS, demonstrated by the presence of hypergammaglobulinemia and autoantibodies against intracellular autoantigens Ro/Sjögren’s syndrome associated autoantigen (SS)A and La/SSB, which are expressed by almost all cell types. The immune complexes formed by autoantibodies lead to innate immune activation and type I interferon (IFN) production, contributing to the chronicity of the disease. Although the pathogenesis of pSS is still unknown, a complex interplay of several factors has been implicated including genetic predisposition, environmental factors, and epigenetic factors [3, 4].

MicroRNAs (miRNAs) are single-stranded, small non-coding (snc)RNAs of 19–25 nucleotides in length that regulate gene expression at the post-transcriptional level [5]. Numerous studies have demonstrated that miRNAs are expressed in different tissues, cell types, and are also present in various biological fluids such as saliva, serum and plasma. Circulating miRNAs can be found in combination with specific carrier proteins or enclosed in different types of vesicles, including exosomes [6, 7]. miRNAs account for 1–5% of the human genome and can negatively regulate expression of at least 30% of protein-coding genes at the post-transcriptional level [8]. A single miRNA can influence many different mRNA targets and conversely, several different miRNAs can bind to a single mRNA target. This regulation can occur at different levels, by mediating mRNA cleavage, repressing mRNA translation or causing mRNA destabilization [9, 10].

miRNAs are involved in the control of immunologic processes such as cell differentiation, proliferation, and apoptosis [11]. As such, miRNAs are thought to play a critical role in autoimmunity and in numerous autoimmune diseases [12]. Recently, several studies in pSS patients demonstrated the dysregulation of specific miRNAs in salivary glands or PBMCs from pSS patients [13, 14]. The expression of miR-768-3p and miR-574 in the salivary glands of patients with pSS is different from those with non-Sjögren’s syndrome and can distinguish subsets of pSS patients with low or high grade salivary gland inflammation [15]. Furthermore, significantly lower expression of miR200b-5p in salivary gland tissue was described in pSS patients with MALT lymphoma compared to pSS patients without history of lymphoma [16].

sncRNAs, including miRNAs, are present in serum and circulating miRNA levels are associated with a range of diseases, including nervous system disorders, metabolic and autoimmune diseases [7, 12, 17]. As serum is easily accessible and collection is relatively easy to standardize, we investigated whether there are differences in the serum levels of 758 sncRNAs between pSS patients and incomplete Sjögren’s Syndrome (iSS) patients or healthy controls (HC). In addition we assessed if serum sncRNA levels can be used to differentiate patients with specific disease features.

## Materials and methods

### Patients and controls

**Fig 1:**
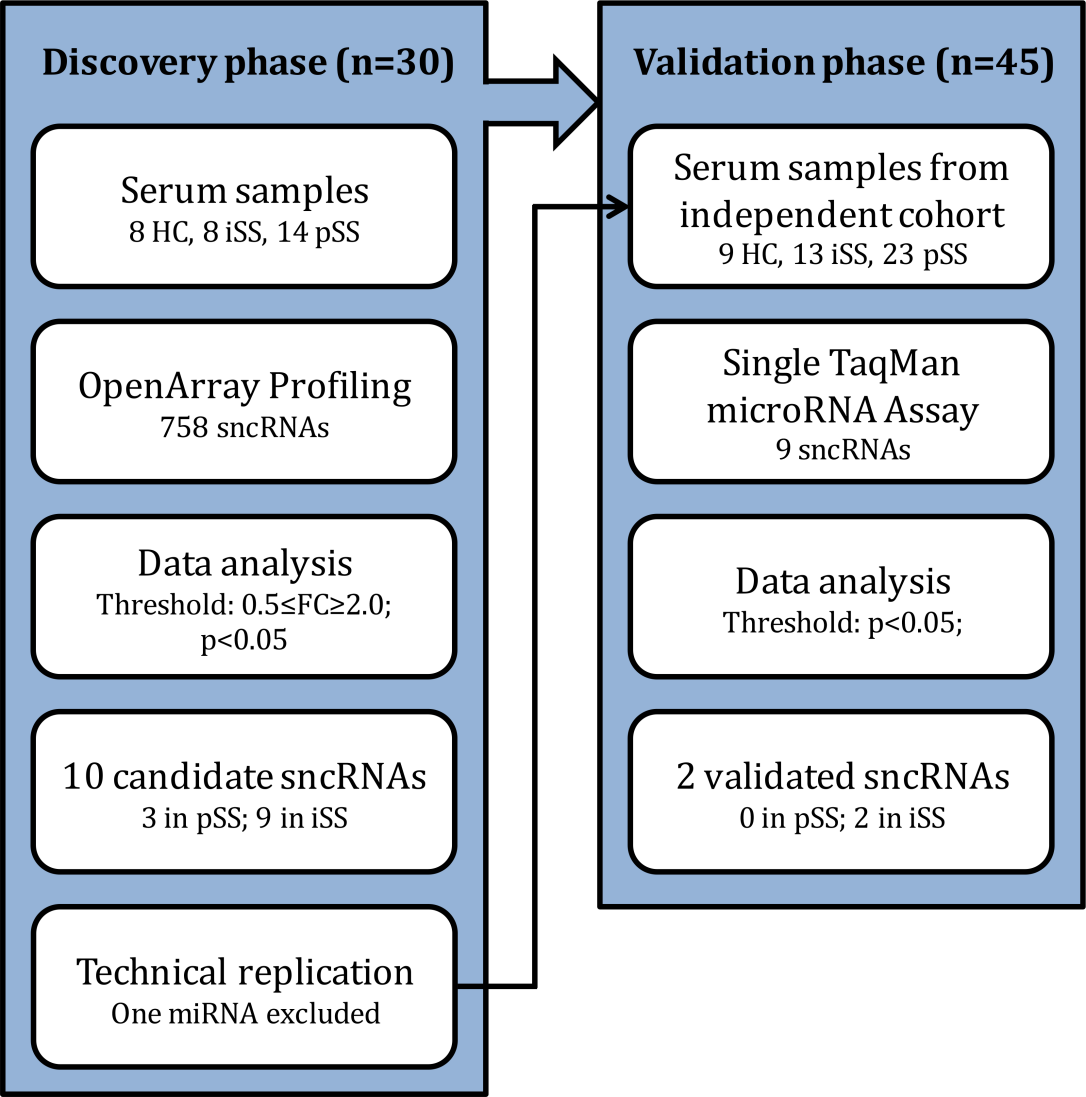
Workflow of discovery and validation approach. sncRNAs were considered to be validated in the validation phase when they reached the threshold of p<0.05 with a difference in the same direction (ie. up/down regulated) as was observed in the discovery phase. HC: healthy control; iSS: incomplete Sjögren’s Syndrome; pSS: primary Sjögren’s syndrome; sncRNA: small non-coding RNA.

Two independent cohorts of patients followed up in the department of rheumatology & clinical immunology at the University Medical Center Utrecht and controls were established: a discovery cohort (14 pSS, 8 iSS, 8 HC) was used to screen the serum abundance of a large panel of 758 sncRNAs, while a validation cohort (23 pSS, 13 iSS, 9 HC) was used to test the reproducibility of the results (Fig 1). Donors were allocated to each cohort random. The patients with pSS were classified according to the AECG criteria [18]. The iSS patients presented with dryness complaints without a known cause, were not clinically considered to have any generalized autoimmune disease including pSS, and did not fulfil the classification criteria for pSS. The study was approved by the ethics committee of the University Medical Center Utrecht. All patients gave their written informed consent in accordance with the declaration of Helsinki. The characteristics of the individuals included in the study are depicted in Table 1.

**Table 1:** Characteristics of the patients and controls enrolled in the study.

	Discovery Cohort (n = 30)				Validation Cohort (n = 45)			
	HC	iSS	pSS	*p-value*	HC	iSS	pSS	*p-value*
N (M/F)	8 [0/8]	8 [0/8]	14 [3/11]	*0*.*149*	9 [1/8]	13 [0/13]	23 [1/22]	*0*.*461*
Age (yr.)	56 [51–67]	42 [25–68]	54 [29–70]	*0*.*229*	45 [29–55]	47 [24–71]	55 [26–77]	*0*.*324*
LFS (foci/4 mm^2^)	-	0.0 [0.0–1.0]	1.9 [1.0–4.0]	***<0*.*001***	-	0.0 [0.0–0.7]	2.0 [1.0–5.0]	***<0*.*001***
ESSDAI	-	-	2.0 [0.0–19]	*-*	-	-	5.0 [0.0–13]	*-*
ESSPRI	-	-	3.7 [2.0–8.8]	*-*	-	-	5.3 [1.0–8.0]	*-*
Schirmer (mm/5 min)	-	3.0 [0.0–24]	5.0 [0.5–25]	*0*.*620*	-	5.8 [1.5–32]	5.5 [0.0–30]	*0*.*483*
ANA (no. positive [%])	-	0 [0%]	10 [71%]	***0*.*002***	-	7 [54%]	19 [86%]	***0*.*050***
SSA (no. positive [%])	-	3 [38%]	8 [57%]	*0*.*659*	-	4 [31%]	18 [78%]	***0*.*011***
SSB (no. positive[%])	-	0 [%]	4 [29%]	*0*.*254*	-	0 [0%]	12 [52%]	***0*.*002***
RF (no. positive [%])	-	0 [%]	5 [42%]	*0*.*114*	-	1 [10%]	9 [47%]	*0*.*098*
IFN-score	2.94 [-2.0–7.0]	-	8.84 [-5.0–14.9]	*0*.*124*	-1.59 [-3.5–4.7]	-	10.7 [-5.1–17.8]	***0*.*048***
Serum IgG (g/L)	-	10 [6.8–17]	14 [8.3–30]	*0*.*070*	-	13 [6.5–15]	15 [5.6–33]	*0*.*061*
ESR (mm/hour)	-	12 [4.0–17]	11 [5.0–36]	*0*.*645*	-	13 [2.0–29]	14 [3.0–63]	*0*.*384*
CRP (mg/L)	-	1.9 [0.0–4.0]	1.0 [0.0–8.0]	*0*.*244*	-	1.0 [0.0–9.3]	1.2 [0.0–13]	*0*.*834*
C3 (g/L)	-	1.1 [0.6–1.7]	1.1 [0.7–1.3]	*0*.*578*	-	1.2 [0.8–1.5]	1.0 [0.8–1.4]	***0*.*029***
C4 (g/L)	-	0.3 [0.2–0.4]	0.3 [0.0–0.3]	*0*.*425*	-	0.3 [0.2–0.4]	0.2 [0.1–0.4]	*0*.*075*
Not treated (no. [%])	-	7 [88%]	11 [79%]	*>0*.*999*	-	10 [77%]	15 [65%]	*0*.*708*
Only HCQ (no. [%])	-	1 [12%]	1 [7%]	*>0*.*999*	-	2 [15%]	3 [13%]	*>0*.*999*
Other (no. [%])	-	0 [0%]	2 [14%]	*0*.*515*	-	1 [8%]	5 [22%]	*0*.*385*

Values are Median [Range] unless stated otherwise. Groups were compared per cohort using Kruskall Wallis test, Fisher’s exact test or Mann-Whitney U test where appropriate. Significant differences (p<0.05) are depicted in bold. HC: Healthy control; iSS: incomplete Sjögren’s syndrome; pSS: primary Sjögren’s syndrome; LFS: Lymphocyte focus score; ESSDAI: EULAR Sjögren’s syndrome disease activity index; ESSPRI: EULAR Sjögren’s syndrome patient reported index; ANA: Anti-nuclear antibodies; SSA: Anti-SSA/Ro; SSB: Anti-SSB/La; RF: Rheumatoid Factor; ESR: Erythrocyte sedimentation rate; CRP: C-reactive protein, HCQ: Hydroxychloroquine. Other treatment group includes Azathioprine, alone or in combination with Prednisone (n = 5); Mesalazine (n = 1); HCQ in combination with Prednisone (n = 1); Prednisone (n = 1).

### Serum RNA preparation

Fresh blood samples were collected in Vacutainer SSTII Advance tubes (BD Biosciences, Franklin Lakes, NJ, USA). Serum was collected as per manufacturer’s instructions, snap frozen in liquid nitrogen and stored at -80°C until further use. RNA was extracted from 240uL of serum using the miRcury RNA isolation kit for biofluids (Exiqon, Vedbaek, Denmark). At the first step of extraction, 300pg of a synthetic miRNA (Arabidopsis thaliana ath-miR-159a) was added to each sample as a spike-in.

### sncRNA profiling array

sncRNA profiling in the discovery cohort was performed on the OpenArray platform (Life Technologies, Carlsbad, CA, USA). Profiling was performed as previously described [19]. Data were analyzed using ExpressionSuite software (Life Technologies), using the relative threshold cycle (Crt) and the comparative threshold cycle method. Data were normalized using both the global mean normalization approach [20] and normalization by ath-miR-159a spike-in [21]. Low expressed sncRNAs (Crt higher than 27) were set at 27; samples with an amplification score lower than 1.24 were excluded from all analyses. Relative expression was calculated by dividing the Crt of each sample by that of a random sample in the healthy control group, which was set at 1. Differences in sncRNA expression between the groups in the discovery cohort using global mean normalization with a FC difference of ≤0.5 or ≥2.0 at an uncorrected p-value of p<0.05 between any of the groups were selected for validation analysis.

### sncRNA validation

For biological validation, miRNA-specific TaqMan RT-qPCR was performed on the samples from the validation cohort. In the same experiment, all samples from the discovery cohort were re-measured for technical replication and to allow the merging of the data for studying associations with clinical parameters and clustering analysis. To this end, the following sncRNA assays were ordered from Life Technologies: U6-snRNA (ID 001973), hsa-miR-23a-3p (ID 000399), hsa-miR-223-5p (ID 002098), hsa-miR-661 (ID 001606), hsa-miR-143-3p (ID 002249), hsa-miR-342-3p (ID 002260), hsa-miR-150-5p (ID000473), hsa-miR-140-5p (ID 001187), hsa-miR-29c-3p (ID 000587), hsa-miR-212-3p (ID 000515) and for the exogenous control ath-miR-159a (ID 000338). From 2.5 uL of serum RNA, cDNA was synthesized by using the individual miRNA-specific RT primers contained in the TaqMan miRNA assays in the presence of 3.3 U/uL MultiScribe RT enzyme (Life Technologies), by using the following thermal cycler conditions: 10 min at 4°C, 30 min at 16°C, 30 min at 42°C, 5 min at 85°C. miRNA levels were quantified in duplicate from 3uL of cDNA using TaqMan fast advance master mix and miRNA-specific primers from the TaqMan miRNA assays, using these amplification conditions on the Quantstudio 12k Real-Time PCR system (Life Technologies): 2 min at 50°C, 20 sec at 95°C, followed by 40 cycles of 1 sec at 95°C, 20 sec at 60°C. sncRNA expression was calculated after normalization by ath-miR-159a spike-in (ΔCt = Ct mean target–Ct mean miR-159a). The relative fold change (FC) of each sample was calculated in comparison with the ΔCt mean of the HC group (reference) according to the formula FC = 2^-ΔΔCt^, where ΔΔCt = ΔCt sample—ΔCt reference. Technical replication was considered to be successful if there was a robust correlation between the Ct in the discovery array (Crt) and the Ct in the single-assay RT-qPCR (r>0.5 and p<0.05). Validation was considered successful if the direction of the difference (ie. up/downregulation) was identical to what was observed in the discovery cohort and the difference was significant at an uncorrected p-value of p<0.05.

### Hierarchical clustering

For unsupervised hierarchical clustering, Euclidian distance with complete linkage was used on the FC of sncRNA levels to divide the pSS patients into clusters using the Multi Experiment Viewer online software (http://mev.tm4.org).

### Interferon signature quantification

At the time of blood drawing for serum collection, additional blood was drawn from 13 of the healthy controls and 25 of the pSS patients (randomly selected) to determine the IFN signature. To this end, mononuclear cells were isolated from heparinized peripheral blood by density centrifugation using Ficoll-Paque Plus (GE Healthcare, Uppsala, Sweden). CD14^+^ monocytes were isolated by magnetic-activated cell sorting using CD14^+^ isolation kit (Miltenyi Biotec, Bergisch Gladbach, Germany) according to manufacturer’s instructions. To confirm consistent purity of isolated monocytes, cells were stained with the following monoclonal antibody combination: anti-CD45 Peridinin chlorophyll (clone: HI30; Sony Biotechnology, San Jose, California, USA), anti-CD16 Phycoerythrin (clone: DJ130C; Agilent, Santa Clara, California, USA) and anti-CD14 Fluorescein isothiocyanate (clone: TÜK4; Miltenyi Biotec) and the proportion of CD14^+^ cells within the isolated fraction was measured using Fluorescence associated cell sorting (FACS) and a FACSCanto II flow cytometer (BD Bioscience, San Jose, USA). The purity of the monocyte samples was (median [range]) 98% [90–99%], there were no significant differences in cell purity between the groups. Cells were lysed in RLTPlus buffer (Qiagen, Venlo, Netherlands) supplemented with 1% of Beta-mercaptoethanol. Total RNA was purified using AllPrep DNA/RNA/miRNA Universal Kit (Qiagen) according to the manufacturer’s instructions. RNA concentration was assessed with Qubit RNA Kit (Life Technologies). To determine the IFN-score, the relative expression of 5 Interferon-induced genes (IFI44L, IFI44, IFIT3, LY6E and MX1) was assessed as previously described [22] relative to the expression in the healthy control group, using the Quantstudio system (Life Technologies).

### Statistics

Statistical analyses were performed using GraphPad Prism software version 6.02 (GraphPad, Lo Jolla, CA, USA) and IBM SPSS version 21 (IBM Corp, Armonk, NY. USA). Mann-Whitney U-test was used to compare groups in the discovery and validation analyses, without correction for multiple testing. Kruskal-Wallis H test with post-hoc Dunn’s test of multiple comparisons was used to compare clusters. For correlations, Spearman’s rho was used and p-values were corrected for multiple testing using B&H FDR. Fisher’s exact test was used to compare categorical variables. Two-sided testing was performed for all analyses. Differences and correlations were considered statistically significant at p<0.05.

## Results

### Discovery of sncRNAs using OpenArray-based miRNA profiling

OpenArray-based analysis of 758 sncRNAs was performed in the serum of pSS patients, iSS patients, and healthy controls (HC) from the discovery cohort (n = 30). All differences in sncRNA abundance between the groups that were significant when using spike-in normalization were also significant when using global mean normalization (S1 Table). We based our further analysis on the data from the global mean normalization to be as inclusive as possible and because this methodology is considered to be the gold standard [23, 24]. When global mean normalization was used, the levels of three sncRNAs were significantly different in pSS patients and nine sncRNAs were significantly different in patients with iSS as compared to HC. Two of these sncRNAs (U6-snRNA and miR-29c-3p) were different in both patient groups compared to HC. There were no differences in sncRNA abundance between pSS and iSS patients that met the set thresholds (Table 2).

**Table 2:** Results from discovery and validation cohort analyses.

	**Discovery Cohort (n = 30)**		**Validation Cohort (n = 45)**	
	iSS vs HC	pSS vs HC	iSS vs HC	pSS vs HC
miR-29c-3p	**5.308 (0.004)**	**5.659 (<0.0001)**	1.070 (0.744)	1.009 (0.910)
U6-snRNA	**4.280 (0.003)**	**2.461 (0.016)**	**2.388 (0.020)**	1.962 (0.490)
miR-23a-3p	**2.718 (0.007)**	2.124 (0.123)	0.706 (0.009)	0.881 (0.305)
miR-661	**2.609 (0.021)**	1.978 (0.019)	**1.743 (0.007)**	1.861 (0.312)
miR-150-5p	**2.168 (0.028)**	1.265 (0.353)	1.247 (0.558)	1.162 (0.405)
miR-143-3p	**2.088 (0.007)**	1.692 (0.016)	0.903 (0.595)	1.080 (0.993)
miR-140-5p	**2.062 (0.021)**	1.364 (0.182)	0.980 (0.632)	0.900 (0.300)
miR-223-5p	**2.001 (0.049)**	1.141 (0.642)	1.123 (0.618)	1.355 (0.790)
miR-342-3p	**2.384 (0.001)**	1.496 (0.309)	1.081 (0.683)	1.013 (0.516)
miR-212-3p	0.417 (0.232)	**0.129 (0.002)**	-	-

Results are expressed as mean FC (p-value). Differences between groups that met the threshold for the corresponding analysis (for discovery: FC difference of ≤0.5 or ≥2.0 at p-value of p<0.05; for validation: FC difference in same direction as seen in discovery at p<0.05) are indicated in bold. Mann–Whitney U test was used to test all comparisons. No differences that met the set thresholds were observed between pSS and iSS in the discovery cohort. miR-212-3p was not technically replicated and therefore was not included in the validation cohort analysis.

For technical replication, we measured the ten differentially expressed sncRNAs in all of the donors included in the discovery cohort using single-assay RT-qPCR. Nine out of these ten sncRNAs (all but miR-212-3p) showed a robust correlation between the relative expression measured in the OpenArray and in the single-assay RT-qPCR, and were therefore included in the validation phase (S2 Table).

### Validation of the selected sncRNAs in an independent cohort

The nine sncRNAs that were technically replicated were measured in an independent validation cohort (n = 45) using single-assay RT-qPCR. None of the sncRNAs that were identified as differentially expressed in the pSS group in the discovery cohort were validated. Of the sncRNAs that were differentially expressed in the iSS group compared to HC in the discovery cohort, two were validated: U6-snRNA and miRNA-661 (Table 2).

### Serum sncRNA expression is associated with laboratory disease parameters in pSS patients

**Fig 2:**
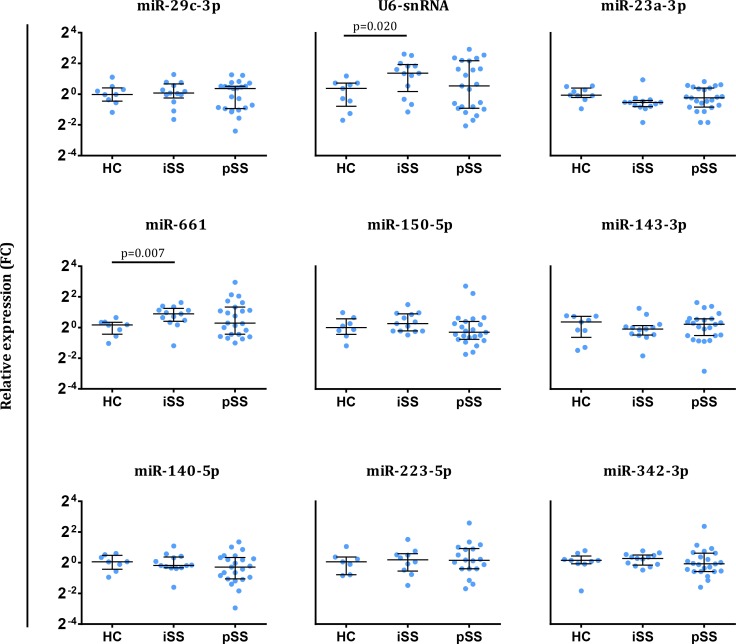
RT-qPCR data of all nine sncRNAs included in the validation phase. Serum sncRNAs were measured using single Taqman qRT-PCR in the validation cohort (n = 45). ΔCt per sample was calculated using the expression of an exogenous spiked-in Arabidopsis thaliana miRNA to correct for technical variation. The relative expression of each sample was calculated as fold change (FC) in comparison with the ΔCt mean of the HC group in the respective cohort. Medians ± IQR are shown.

Although the sncRNAs included in the validation phase were not differentially expressed in pSS patients within the validation cohort, we observed a large spread in expression for all nine of the sncRNAs within this group (Fig 2). This observation prompted us to investigate whether sncRNA abundance was related to clinical or laboratory parameters within the pSS patients. For this, we used the single-assay RT-qPCR data from both cohorts. None of the sncRNAs showed a significant association with demographic data (sex, age) or clinical features (ESSDAI, ESSPRI, Schirmer), yet several of them showed correlations with laboratory parameters including LFS (Table 3). Interestingly, many of the parameters known to be associated with high disease activity (i.e. low C3/C4, decreased leukocyte count, high lymphocytic focus score) were negatively correlated with the abundance of the sncRNAs investigated (Table 3). In addition, pSS patients who are positive for anti-Ro (SSA) and/or anti-La (SSB) showed decreased expression of several of the sncRNAs when compared to the antibody-negative pSS patients (S3 Table). Thus, the spread in sncRNA expression in the pSS group is related to their heterogeneity in disease parameters.

**Table 3:** Correlations between serum sncRNA levels and disease parameters in pSS patients.

		**C3 (g/L)**	**C4 (g/L)**	**sIgG (g/L)**	Leukocytes (*10^**9**^**/L)**	Lymphocytes (*10^**9**^**/L)**	**LFS** **(foci/4mm**^**2**^**)**	**IFN** **score**
miR-29c-3p	*ρ*	0.372	**0.415**	**-0.429**	**0.520**	0.183	-0.282	**-0.502**
	p	0.050	**0.042**	**0.038**	**0.009**	0.387	0.147	**0.039**
U6-snRNA	*ρ*	0.381	**0.400**	**-0.432**	**0.697**	0.226	-0.208	**-0.545**
	p	0.050	**0.047**	**0.038**	**0.000**	0.285	0.269	**0.029**
miR-23a-3p	*ρ*	**0.463**	0.268	**-0.401**	**0.382**	0.111	**-0.393**	-0.442
	p	**0.029**	0.171	**0.046**	**0.049**	0.602	**0.049**	0.050
miR-661	*ρ*	0.374	0.236	**-0.520**	**0.605**	0.112	-0.245	**-0.675**
	p	0.050	0.218	**0.009**	**0.003**	0.602	0.204	**0.004**
miR-150-5p	*ρ*	0.253	**0.534**	-0.291	**0.434**	**0.483**	**-0.387**	-0.250
	p	0.190	**0.009**	0.131	**0.038**	**0.038**	**0.049**	0.269
miR-143-3p	*ρ*	0.378	0.229	**-0.491**	**0.527**	0.070	-0.372	**-0.491**
	p	0.050	0.229	**0.017**	**0.009**	0.731	0.050	**0.042**
miR-140-5p	*ρ*	**0.403**	0.346	**-0.422**	**0.543**	0.103	-0.264	**-0.468**
	p	**0.047**	0.071	**0.039**	**0.009**	0.621	0.176	**0.048**
miR-223-5p	*ρ*	0.275	0.327	-0.343	0.207	0.163	-0.410	-0.302
	p	0.190	0.122	0.095	0.296	0.498	0.050	0.207
miR-342-3p	*ρ*	0.292	**0.386**	-0.222	0.332	0.406	**-0.421**	-0.072
	p	0.133	**0.049**	0.233	0.079	0.056	**0.041**	0.731

Spearman’s correlation coefficients (*ρ*) and B&H FDR-corrected p-values are shown. sIgG: serum immunoglobulin G; LFS: lymphocytic focus score. Correlations that are significant at p<0.05 are depicted in bold.

### Hierarchical clustering

**Fig 3:**
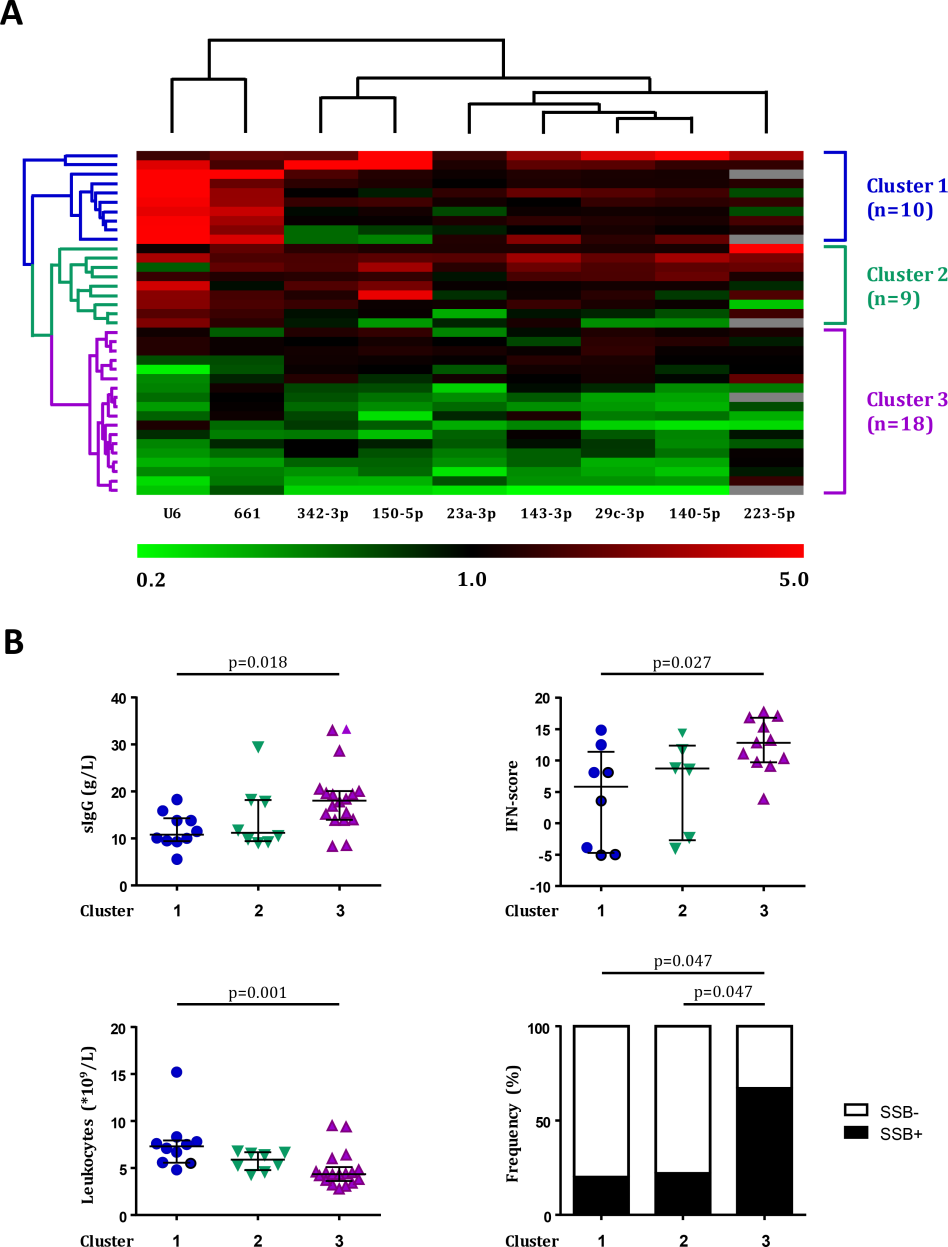
pSS patients with increased B cell hyperactivity can be identified using hierarchical clustering of serum sncRNA expression levels. Nine sncRNAs were selected based on their differential expression in the discovery array and subsequent technical replication. These sncRNAs were measured using single-assay RT-qPCR in samples from both cohorts (n = 75). Unsupervised hierarchical clustering was performed on the expression of the nine selected sncRNAs in all 37 pSS patients. Grey fields depict unavailable data points (A). Clinical parameters and frequency of positivity for anti-La (SSB) autoantibodies were compared between the three clusters (B). The patients in each cluster were compared using Kruskal-Wallis H test with post-hoc Dunn’s test of multiple comparisons and Fisher’s exact test. For dot plots, medians ± IQR are shown.

Since each sncRNA was associated with a distinct set of laboratory parameters, we next investigated whether specific patterns of sncRNA expression could distinguish subsets of patients with a certain disease phenotype within the pSS group. To this end, unsupervised hierarchical clustering was used to group the most similar pSS patients on the basis of their expression of each of the nine sncRNAs measured by single-assay RT-qPCR. This allowed the identification of three distinct clusters of patients based on different sncRNA patterns (Fig 3A). Clustering analysis showed that one group of patients (cluster 3) had an overall decreased expression of all nine sncRNAs in their serum, while two groups (clusters 1 and 2) had higher serum levels for at least one of the measured miRNAs. Comparison of clinical parameters between clusters showed that patients in cluster 3 presented with higher serum IgG and IFN-score, as well as decreased leukocyte counts compared to cluster 1. In addition, an increased frequency of patients in this cluster was positive for anti-La (SSB) (Fig 3B).

## Discussion

In the present study, we investigated whether serum sncRNAs can be used to distinguish pSS patients from iSS patients and HC. Using two independent cohorts with patients and controls, we were unable to identify any sncRNAs that reproducibly differ between pSS patients and patients with iSS or HC. However, we did show that circulating sncRNA levels reflect disease parameters in pSS patients and can be used to distinguish pSS patients with higher markers of B cell hyperactivity.

We chose to measure circulating miRNAs in serum, as it is easily accessible and its preparation is well standardized. Measurements of miRNAs in plasma may yield different results, as a recent study showed that around 6% of studied miRNAs show differences in expression between the 2 fluids [25]. Future studies need to be conducted to investigate whether more clear differences in miRNA levels can be found in plasma from pSS patients. However, levels of eight of the nine sncRNAs included in our validation phase (all but miR-661) were previously compared between serum and plasma and none of them exhibited significant differences [25]. As such, these miRNAs should be similarly expressed in plasma measurements.

Our discovery-validation approach allowed us to measure a large number of sncRNAs in the discovery cohort and follow nine of these up in the validation cohort. Technical replication showed that the differences found in the discovery cohort were not artifacts of the array. In addition, we compared two methods of data normalization for the discovery cohort to ensure optimal data analysis. We chose to use global mean normalization as it was the most inclusive and appropriately corrects for limitations intrinsic to the qPCR methodology [20, 23]. As such, any serum sncRNAs that were included in the array and are robustly dysregulated in pSS patients compared to iSS or HC should have been identified here. Using this approach, we validated that two sncRNAs, U6-snRNA and miR-661, are increased in iSS patients compared to HC. To our knowledge, these sncRNAs have not previously been described in any autoimmune disease and future studies on the function of these sncRNAs should clarify what their role is in the disease. Although the iSS patients who were studied presented with mild local and systemic parameters of inflammation, these features may explain the increased sncRNA levels. In-line with this hypothesis, data showed an association between increased circulating U6-sncRNA and markers of inflammation in a range of inflammatory conditions [26].

However, the lack of differences between pSS patients and iSS patients or HC in sncRNAs can be largely attributed to the heterogeneity of this group. The expression of the nine sncRNAs measured in the validation phase was strongly overlapping between pSS patients, iSS patients and HC. As our analyses show an association between serum sncRNA levels and several biological disease parameters, the large variation in the pSS group seems to be related to differences in markers of inflammation. In particular, autoantibody presence was an important parameter in this regard, as a range of sncRNAs showed significant differences between autoantibody positive and negative pSS patients. As such, the increased prevalence of SSA and SSB positivity in the validation cohort compared to the discovery cohort, although not statistically significant, may have contributed to the lack of validated targets. However, both cohorts presented with an autoantibody presence that is within acceptable range to those reported in very large cohorts of pSS patients [27].

Within the nine sncRNAs measured in the validation phase, we observed an overall trend of increased expression in the pSS patients from clusters 1 and 2 compared to the patients in cluster 3. The pSS patients in cluster 3 showed an overall decrease in serum levels of the measured sncRNAs and presented with more pronounced autoimmune activity, including increased B cell hyperactivity, as measured by sIgG and autoantibody positivity, and a higher IFN-score. The IFN-score was previously shown to correlate with the disease activity and autoantibody presence in pSS patients [22], which is in-line with the increased serum IgG and SSB-positivity we observed in the patients in this cluster. The association of lower sncRNA levels with higher parameters of B cell hyperactivity may be explained by a change in the composition of circulating B cell pool. In line with this hypothesis, three of miRNAs analyzed in the validation phase (miR-150-5p, miR-223-5p, and miR-342-3p) are highly expressed by naïve B cells while their expression is down-regulated upon B cell activation. In addition, these miRNAs were implicated in the regulation of B cell differentiation [28]. As such, this set of miRNAs may be involved in the increased B cell hyperactivity observed in the patients of cluster 3. Alternatively, the lower sncRNA levels observed in cluster 3 may be a reflection of changes in the composition of circulating cells, as these patients also have a decreased leukocyte count. Possibly, the decreased levels of sncRNAs can be explained by migration of the leukocytes responsible for the production of these sncRNAs to sites of inflammation. This is supported by the correlation of leukocyte counts with the expression of the majority of the sncRNAs measured in the validation phase in the pSS group as a whole. In line with this, levels of circulating miRNAs that are expressed by leukocyte subsets correlate with the presence of these cells in the blood [29].

## Conclusions

In conclusion, we validated increased expression of two serum sncRNAs in iSS patients compared with HC, U6-snRNA and miR-661, but did not find any differences in serum sncRNA levels between pSS patients and iSS patients or HC. Furthermore, we show that the heterogeneity in sncRNA expression within the pSS patients is associated with differences in clinical and laboratory parameters. Moreover, pSS patients with a higher IFN score and signs of increased B cell hyperactivity can be distinguished on the basis of overall lower expression levels of the sncRNAs studied. These lower serum sncRNA levels may be related to migration of the miRNA-producing cells from the circulation towards the site of inflammation. In addition, as several of these miRNAs are implicated in B cell activation and differentiation, they may play a role in the more pronounced B cell hyperactivity observed in these patients.

## Supporting information

S1 Table Comparison between global mean and spike-in normalization in the discovery cohort. Results are expressed as mean FC. Differences between groups that met the threshold for the corresponding analysis (FC difference of ≤0.5 or ≥2.0 at p-value of p<0.05) are indicated in bold. Mann–Whitney U test was used to test all comparisons.
(DOCX)

S2 Table Correlation between array and single RT-qPCR results in the discovery cohort. Correlation between Crt in profiling array and CT measured with single-assay Taqman RT-qPCR in patients and controls from the discovery cohort (n = 30). Spearman’s correlation coefficients (*ρ*) and p-values are shown. Correlations that are significant at p<0.05 are depicted in bold.
(DOCX)

S3 Table Differences between SSA/SSB positive and negative pSS patients in circulating sncRNA abundance. sncRNAs were measured using RT-qPCR in all pSS patients from the discovery and validation cohort (n = 37). Fold changes (FC) were calculated as compared to the mean of the healthy control group in the corresponding cohort. Results are expressed in FC as median [range]. Statistically significant differences (Mann–Whitney U test) between autoantibody positive and negative pSS patients are indicated in bold. SSA: anti-Ro/Sjögren’s syndrome antigen A; SSB: anti-La/Sjögren’s syndrome antigen B. * and ** depict significant differences at p<0.05 and p<0.01 respectively.
(DOCX)
