# Supplementary material for: Circulating small non-coding RNAs reflect IFN status and B cell hyperactivity in patients with primary Sjögren’s syndrome
Source: PLoS One. 2018 Feb 15;13(2):e0193157. doi: 10.1371/journal.pone.0193157 (PMC5814054; doi:10.1371/journal.pone.0193157)
Supplement: S1 Table — Results are expressed as mean FC. Differences between groups that met the threshold for the corresponding analysis (FC difference of ≤0.5 or ≥2.0 at p-value of p<0.05) are indicated in bold. Mann–Whitney U test was used to test all comparisons. (DOCX) [file pone.0193157.s001.docx]

**S1 Table. Comparison between global mean and spike-in normalization in the discovery cohort.**

|  |  | Global mean normalization | |  | Spike-in normalization | |
| --- | --- | --- | --- | --- | --- | --- |
|  |  | iSS vs HC | pSS vs HC |  | iSS vs HC | pSS vs HC |
| miR-29c-3p |  | **5.308 (0.004)** | **5.659 (<0.0001)** |  | **9.686 (0.003)** | **7.531 (0.0001)** |
| U6-snRNA |  | **4.280 (0.003)** | **2.461 (0.016)** |  | **3.848 (0.003)** | 2.634 (0.106) |
| miR-23a-3p |  | **2.718 (0.007)** | 2.124 (0.123) |  | **2.284 (0.007)** | 2.079 (0.110) |
| miR-661 |  | **2.609 (0.021)** | 1.978 (0.019) |  | 2.146 (0.161) | 2.000 (0.165) |
| miR-150-5p |  | **2.168 (0.028)** | 1.265 (0.353) |  | 1.936 (0.161) | 1.356 (0.441) |
| miR-143-3p |  | **2.088 (0.007)** | 1.692 (0.016) |  | 1.698 (0.015) | 1.635 (0.095) |
| miR-140-5p |  | **2.062 (0.021)** | 1.364 (0.182) |  | 1.696 (0.159) | 1.356 (0.330) |
| miR-223-5p |  | **2.001 (0.049)** | 1.141 (0.642) |  | 1.648 (0.336) | 1.144 (0.971) |
| miR-342-3p |  | **2.384 (0.001)** | 1.496 (0.309) |  | 1.736 (0.123) | 1.235 (0.616) |
| miR-212-3p |  | 0.417 (0.232) | **0.129 (0.002)** |  | 0.342 (0.195) | **0.135 (0.002)** |

Results are expressed as mean FC. Differences between groups that met the threshold for the corresponding analysis (FC difference of ≤0.5 or ≥2.0 at p-value of p<0.05) are indicated in bold. Mann–Whitney U test was used to test all comparisons.
