# Supplementary material for: Circulating small non-coding RNAs reflect IFN status and B cell hyperactivity in patients with primary Sjögren’s syndrome
Source: PLoS One. 2018 Feb 15;13(2):e0193157. doi: 10.1371/journal.pone.0193157 (PMC5814054; doi:10.1371/journal.pone.0193157)
Supplement: S2 Table — Correlation between Crt in profiling array and CT measured with single-assay Taqman RT-qPCR in patients and controls from the discovery cohort (n = 30). Spearman’s correlation coefficients (ρ) and p-values are shown. Correlations that are significant at p<0.05 are depicted in bold. (DOCX) [file pone.0193157.s002.docx]

**S2 Table. Correlation between array and single RT-qPCR results in the discovery cohort.**

|  |  | Correlation | |
| --- | --- | --- | --- |
|  |  | *ρ* | p |
| miR-29c-3p |  | **0.9035** | **<0.0001** |
| U6-snRNA |  | **0.8545** | **<0.0001** |
| miR-23a-3p |  | **0.7044** | **<0.0001** |
| miR-661 |  | **0.5704** | **0.0012** |
| miR-150-5p |  | **0.9547** | **<0.0001** |
| miR-143-3p |  | **0.8645** | **<0.0001** |
| miR-140-5p |  | **0.6089** | **0.0005** |
| miR-223-5p |  | **0.8976** | **<0.0001** |
| miR-342-3p |  | **0.7783** | **<0.0001** |
| miR-212-3p |  | 0.0 | >0.999 |

Correlation between Crt in profiling array and CT measured with single-assay Taqman RT-qPCR in patients and controls from the discovery cohort (n=30). Spearman’s correlation coefficients (*ρ*) and p-values are shown. Correlations that are significant at p<0.05 are depicted in bold
