# Supplementary material for: Circulating small non-coding RNAs reflect IFN status and B cell hyperactivity in patients with primary Sjögren’s syndrome
Source: PLoS One. 2018 Feb 15;13(2):e0193157. doi: 10.1371/journal.pone.0193157 (PMC5814054; doi:10.1371/journal.pone.0193157)
Supplement: S3 Table — sncRNAs were measured using RT-qPCR in all pSS patients from the discovery and validation cohort (n = 37). Fold changes (FC) were calculated as compared to the mean of the healthy control group in the corresponding cohort. Results are expressed in FC as median [range]. Statistically significant differences (Mann–Whitney U test) between autoantibody positive and negative pSS patients are indicated in bold. SSA: anti-Ro/Sjögren’s syndrome antigen A; SSB: anti-La/Sjögren’s syndrome antigen B. * and ** depict significant differences at p<0.05 and p<0.01 respectively. (DOCX) [file pone.0193157.s003.docx]

**S3 Table. Differences between SSA/SSB positive and negative pSS patients in circulating sncRNA abundance.**

|  |  | SSA | |  | SSB | |
| --- | --- | --- | --- | --- | --- | --- |
|  |  | negative | positive |  | negative | positive |
| miR-29c-3p |  | **1.5 [0.5-4.5]** | **1.3* [0.2-2.4]** |  | 1.4  [0.5-4.5] | 1.1  [0.2-2.4] |
| U6-snRNA |  | 1.3 [0.7-5.9] | 1.6 [0.2-7.6] |  | **2.4**  **[0.6-6.6]** | **0.6***  **[0.2-7.6]** |
| miR-23a-3p |  | 1.2  [0.6-2.4] | 0.9 [0.3-1.5] |  | 0.9  [0.3-2.4] | 0.7  [0.3-1.5] |
| miR-661 |  | 2.2  [0.6-7.8] | 1.2  [0.5-4.4] |  | **2.2**  **[0.6-7.8]** | **0.9****  **[0.5-4.4]** |
| miR-150-5p |  | **1.6**  **[0.4-6.4]** | **0.9***  **[0.3-6.5]** |  | 1.3  [0.3-6.4] | 0.8  [0.3-6.5] |
| miR-143-3p |  | **1.5**  **[0.7-3.5]** | **1.0***  **[0.1-3.1]** |  | **1.5**  **[0.6-3.5]** | **1.0***  **[0.1-3.1]** |
| miR-140-5p |  | 1.4  [0.5-5.4] | 0.9  [0.1-2.7] |  | 1.3  [0.4-5.4] | 0.8  [0.1-2.6] |
| miR-223-5p |  | **2.3**  **[0.8-6.0]** | **1.1***  **[0.3-2.5]** |  | **1.5**  **[0.4-6.0]** | **1.0***  **[0.3-2.5]** |
| miR-342-3p |  | 1.6  [0.6-2.5] | 1.0  [0.3-5.2] |  | 1.3  [0.5-2.5] | 1.0  [0.3-5.2] |

sncRNAs were measured using RT-qPCR in all pSS patients from the discovery and validation cohort (n=37). Fold changes (FC) were calculated as compared to the mean of the healthy control group in the corresponding cohort. Results are expressed in FC as median [range]. Statistically significant differences (Mann–Whitney U test) between autoantibody positive and negative pSS patients are indicated in bold. SSA: anti-Ro/Sjögren’s syndrome antigen A; SSB: anti-La/Sjögren’s syndrome antigen B. * and ** depict significant differences at p<0.05 and p<0.01 respectively.
